# IκB kinase promotes Nrf2 ubiquitination and degradation by phosphorylating cylindromatosis, aggravating oxidative stress injury in obesity-related nephropathy

**DOI:** 10.1186/s10020-021-00398-w

**Published:** 2021-10-28

**Authors:** Yin-Yin Chen, Han Hong, Yu-Ting Lei, Jia Zou, Yi-Ya Yang, Li-Yu He

**Affiliations:** 1 Department of Nephrology, The Second Xiangya Hospital of Central South University, Hunan Key Laboratory of Kidney Disease and Blood Purification, No. 139 people’s Middle Road, Changsha, 410011 Hunan People’s Republic of China; 2 Department of Nephrology, Hunan Provincial People’s Hospital, The First Affiliated Hospital of Hunan Normal University, Changsha, 410000 Hunan People’s Republic of China; 3 Changsha Clinical Research Center for Kidney Disease, Changsha, 410000 Hunan People’s Republic of China; 4 Hunan Clinical Research Center for Chronic Kidney Disease, Changsha, 410000 Hunan People’s Republic of China

**Keywords:** IKK, CYLD, Nrf2, Oxidative stress, Phosphorylation, ROS

## Abstract

**Background:**

Obesity-related nephropathy (ORN) has become one of the leading causes of end-stage renal disease and has tripled over the past decade. Previous studies have demonstrated that decreased reactive oxygen species production may contribute to improving ORN by ameliorating oxidative stress injury. Here, IκB kinase (IKK) was hypothesized to inactivate the deubiquitination activity of cylindromatosis (CYLD) by activating the phosphorylation of CYLD, thus promoting the ubiquitination of NF-E2-related factor 2 (Nrf2) and further aggravating oxidative stress injury of the kidney in ORN. This study was aimed to confirm this hypothesis.

**Methods:**

Haematoxylin and eosin (HE), periodic acid-Schiff (PAS) and Oil Red O staining were performed to assess histopathology. Dihydroethidium (DHE) staining and MDA, SOD, CAT, and GSH-PX assessments were performed to measure reactive oxygen species (ROS) production. Immunohistochemical (IHC) staining, qRT–PCR and/or western blotting were performed to assess the expression of related genes. JC-1 assays were used to measure the mitochondrial membrane potential (ΔΨm) of treated HK-2 cells. Co-immunoprecipitation experiments (Co-IP) were used to analyse the interaction between CYLD and Nrf2 in ORN.

**Results:**

ORN in vivo and in vitro models were successfully constructed, and oxidative stress injury was detected in the model tissues and cells. Compared with the control groups, the phosphorylation level of CYLD increased while Nrf2 levels decreased in ORN model cells. An IKK inhibitor reduced lipid deposition, ROS production, CYLD phosphorylation levels and ΔΨm in vitro, which were reversed by knockdown of CYLD. Nrf2 directly bound to CYLD and was ubiquitinated in ORN cells. The proteasome inhibitor MG132 activated the Nrf2/ARE signalling pathway, thereby reversing the promoting effect of CYLD knockdown on oxidative stress.

**Conclusion:**

IKK inactivates the deubiquitination activity of CYLD by activating the phosphorylation of CYLD, thus promoting the ubiquitination of Nrf2 and further aggravating oxidative stress injury of the kidney in ORN. This observation provided a feasible basis for the treatment of kidney damage caused by ORN.

## Background

Obesity has become a serious public health problem as prevalence rates rapidly rise worldwide. More than 60% of adults in the United States are overweight or obese (Wang and Beydoun 2007), and the obesity rates in China also continue to soar (Weiwei et al. 2016). In addition to inducing cardiovascular disease and diabetes (Xia et al. 2019; Su and Peng 2020), an increasing number of reports have shown that obesity is also a significant risk factor for kidney damage, namely, obesity-related nephropathy (ORN) (Hsu et al. 2006; Kambham et al. 2001), which has become one of the leading causes of end-stage renal disease. One study demonstrated that ORN has tripled over the past decade (Hu et al. 2020). Although ORN is potentially preventable by choosing a healthy lifestyle and reducing obesity, the pathogenesis of ORN still needs to be explored to find more effective and safe treatments or drugs for ORN.

Although the mechanisms involved in ORN are complex, numerous studies have shown that oxidative stress, which is a characteristic of obesity (Vincent and Taylor 2006; Karam et al. 2017), is one of the leading causes contributing to kidney injury in ORN (Xu et al. 2017; Shi et al. 2020). The imbalance between the increase in reactive oxygen species (ROS) and/or the decrease in antioxidant activity promotes oxidative stress injury to tissues or cells (Tang et al. 2012; Rani et al. 2016). The production of ROS can induce damage to glomeruli and renal tubules, indicating that ROS play an important role in mediating kidney injury and may eventually lead to the development of end-stage renal disease (Jaimes et al. 2010; Habibi et al. 2011; Fernandes et al. 2016). Thus, reducing ROS production to ameliorate oxidative stress injury may be a new therapeutic target for ORN.

As a positive regulator of the human antioxidant response element (ARE), NF-E2-related factor 2 (Nrf2) plays important roles in defence against oxidative stress by driving the expression of antioxidant enzymes, such as haem oxygenase 1 (HO-1) and NAD(P)H-quinone oxidoreductase 1 (NQO1) (Park et al. 2018; Xu et al. 2016; Venugopal and Jaiswal 1996; Wang et al. 2019). Under normal conditions, Nrf2 binds to Kelch-like ECH-associated protein 1 (Keap1) and is then ubiquitinated by the E3 ligase complex (Al-Sawaf et al. 2015). Nrf2 is released from Keap1 under oxidative stress conditions and binds to the promoter of ARE to enhance the transcription of various antioxidant proteases (Bellezza et al. 2018). Therefore, activating Nrf2 to eliminate oxidative stress may be an effective therapeutic target. In addition, a previous study indicated that the Nrf2-Keap1 signalling pathway has protective effects on diabetic nephropathy (Gong et al. 2018), which also involves oxidative stress as the main pathogenic mechanism. Thus, we believe that it is necessary to explore the mechanism of Nrf2 in ORN.

Ubiquitination, controlled by ubiquitin enzymes and deubiquitinating enzymes (DUBs), is an important molecular mechanism that regulates many cellular processes (Hershko and Ciechanover 1998). Cylindromatosis (CYLD) is a DUB that has been reported to mediate the deubiquitination of certain signalling molecules (Trompouki et al. 2003; Kovalenko et al. 2003). However, IκB kinase (IKK) has been reported to induce the phosphorylation of CYLD. Phosphorylation, as a mechanism, temporarily inactivates the deubiquitination activity of CYLD, thereby promoting the ubiquitination of its downstream molecules (Reiley et al. 2005; Hutti et al. 2009). Furthermore, a previous study suggested that CYLD can inhibit Nrf2-mediated antioxidative capacity, thereby enhancing oxidative stress in the heart (Wang et al. 2015), indicating a potential regulatory relationship between CYLD and Nrf2, which may induce oxidative stress. Based on the above observations, we hypothesized that IKK inactivates the deubiquitination activity of CYLD by activating the phosphorylation of CYLD, thus promoting the ubiquitination of Nrf2 and further aggravating oxidative stress injury to the kidney in ORN.

In this study, we observed that IKK promoted renal injury caused by obesity through CYLD phosphorylation and that an IKK inhibitor could alleviate lipid deposition and oxidative stress injury in ORN, which may provide a feasible target for the treatment of kidney damage caused by ORN.

## Methods

### Animal modelling and grouping

Male ob/ob mice (4 weeks, n = 10) and homologous C57BL/6 (n = 10) mice were purchased from the Animal Experiment Center of the Chinese Academy of Sciences (Shanghai, China) and randomly divided into 2 groups: control and ob/ob. All mice were kept in single cages with 12 h light–dark cycles at 25 °C and 50% relative humidity. The ob/ob mice were fed a high-fat diet (protein 16%, fat 60%, carbohydrate 24%), and the control mice were fed a normal diet (protein 23%, fat 12%, carbohydrate 65%). The body weights of all mice were measured every 4 weeks. After 12 weeks, mice were sacrificed, and then kidneys were obtained for further experiments. All experiments were performed according to protocols from the Second Xiangya Hospital of Central South University and approved by the Second Xiangya Hospital of Central South University.

### Cell culture and treatment

Human kidney proximal tubular epithelial cells (HK-2) derived from the normal kidneys of male adults were purchased from the American Type Culture Collection (ATCC, Manassas, VA, USA) and grown in Dulbecco’s modified Eagle’s medium (DMEM)-F12 medium (Thermo Scientific, Waltham, MA, USA) supplemented with 10% foetal bovine serum (FBS, Gibco, NY, USA), penicillin (100 U/ml, Thermo) and streptomycin (100 µg/ml, Thermo) in a humid incubator containing 5% CO_2_ at 37 °C. After reaching 85% confluence, the cells were divided into 2 groups: the control group (treated with complete medium) and the ox-LDL group (ORN model group) (stimulated with oxidized low-density lipoprotein (ox-LDL)). Then, the IKK inhibitor TPCA1, CYLD-specific shRNA plasmid (sh-CYLD), MG132 or their corresponding controls were incubated with treated cells, and the groups were named as follows: ox-LDL + TPCA1, ox-LDL + TPCA1 + sh-NC, ox-LDL + sh-CYLD, ox-LDL + TPCA1 + sh-CYLD, ox-LDL + sh-NC, ox-LDL + MG132, and ox-LDL + sh-CYLD + MG132, ox-LDL + tempol. Tempol (10 μmol/l) was added 30 min prior to the addition of ox-LDL (ox-LDL + tempol). All groups were maintained at 37 °C for further experimentation.

### RNA extraction and quantitative real-time PCR (qRT–PCR) assay

The relative mRNA expression of CYLD, HO-1, NQO1, kidney injury molecule-1 (KIM-1) and neutrophil gelatinase-associated lipocalin (NGAL) was analysed by qRT–PCR. Briefly, the extraction of total RNA from HK-2 cells and mouse urine samples was performed using TRIzol reagent (Invitrogen, Carlsbad, CA, USA) and QIAzol reagent (Qiagen, Hilden, Germany), respectively. Then, RNA quality was assessed using a NanoDrop 2000c (Thermo) according to the manufacturer’s protocols. Next, the RNAs were converted into cDNA using a RevertAid First Strand cDNA Synthesis kit (Thermo), and qRT–PCR was performed on an ABI 7900 system using SYBR Green Real-Time PCR master mixes (Thermo). The PCR parameters were as follows: 90 °C for 45 s followed by 40 cycles of denaturation at 90 °C for 10 s and annealing at 58 °C for 30 s. Primers were synthesized by RiboBio (Guangzhou, Chnia). The quantitative PCR results were calculated using the 2^−ΔΔCt^ method.

### Western blotting 

Total protein was extracted using cell lysate. After the protein concentration was quantified by a Bradford Protein Assay kit (Beyotime Institute of Biotechnology, Haimen, China), the samples were separated by 10% sodium dodecyl sulfate polyacrylamide gel electrophoresis (SDS–PAGE) and transferred to polyvinylidene difluoride (PVDF) membranes (Millipore, MA, USA), which were blocked with 5% skim milk for 1 h at room temperature. Subsequently, the membranes were incubated overnight at 4 °C with primary antibodies against IKK (1:5000, ab178872, Abcam, MA, USA), P-IKK (1:1000, ab38515, Abcam), CYLD (1:2000, ab153698, Abcam), p-CYLD (1:1000, Ser418 #4500, Cell Signaling Technology), Nrf2 (1:1000, ab89443, Abcam), p-Nrf2 (1:10000, ab76026, Abcam), HO-1 (1:2000, ab52947, Abcam), NQO1 (1:10000, ab80588, Abcam), and HA-Ub (1:3000, ab19247, Abcam) and their corresponding secondary antibodies at 37 °C for 1 h. Finally, the protein bands were visualized by ECL reagent (Millipore, Bedford, MA, USA), and β-actin was used as an internal control.

### Biochemical analysis and urinary microalbumin test

Blood samples were collected after anaesthetizing the mice. A blood glucose monitor (ACCU-CHEK, Roche, Germany) was used to determine the blood glucose levels of the mice. Cholesterol and creatine in serum and triglycerides (TGs) in serum and kidney tissues were measured by corresponding ELISA (enzyme-linked immunosorbent assay) kits (Exocell, Philadelphia, PA, USA) according to the manufacturer’s protocols. Urine was collected (mice were singly housed in metabolic cages to enable urine collection) from mice fed a normal or high-fat diet for 10 days. Urinary microalbumin was measured by ELISA kit (Exocell). Blood urea nitrogen (BUN) levels were detected by a BC-2800 Vet Animal Auto Biochemistry Analyzer (Guangzhou Shihai Medical Equipment Co., Ltd., Guangdong, China).

### Morphological analysis of kidney tissue

The obtained mouse kidneys were placed in 10% neutral-buffered formalin fixative at room temperature overnight and embedded in paraffin. Then, 5 µm thick sections were prepared for staining with haematoxylin and eosin (HE) and periodic acid-Schiff (PAS). The changes in histopathology were observed under a fluorescence microscope (Nikon, Tokyo, Japan).

### Oil red O staining

The lipid deposition in mouse kidneys or treated HK-2 cells was measured using Oil Red O staining. In short, samples were stained with Oil Red O after fixation with a 4% paraformaldehyde solution. 30 min later, the samples were counterstained with haematoxylin for 5 min, and then microscopy was used to observe the results.

### Dihydroethidium (DHE) stain

Reactive oxygen species (ROS) production was measured using microfluorimetry detection of the oxidation of DHE to ethidium. Five-micrometer-thick frozen kidney sections or treated cells were washed with PBS and then incubated with DHE for 30 min. Next, an inverted fluorescence microscope was used to observe superoxide formation.

### Measurement of oxidative stress

The MDA levels and the activities of SOD, CAT, and GSH-px in mouse kidneys or treated cells were assayed by ELISA kits according to the manufacturer’s instructions.

### Immunohistochemistry (IHC) staining

Sections of kidney (5-μm-thick) were conventionally dewaxed in xylene and rehydrated in graded alcohol. Subsequently, the sections were heated in an 85–95 °C microwave oven for 2 min in 0.01 M citrate buffer (pH 6.0). After cooling, the sections were incubated with an anti-Nrf2 primary antibody (1:1000, ab89443, Abcam) and an anti-HO-1 primary antibody (1:2000, ab52947, Abcam) at 4 °C overnight and then washed with PBS 3 times. Next, after incubation with the biotinylated secondary antibody for 30 min at room temperature, the sections were stained with freshly prepared 3,3′-diaminobenzidine (DAB), followed by counterstaining with haematoxylin. After being sealed in neutral resin, the sections were observed under a microscope.

### Mitochondrial membrane potential (MMP, ΔΨm) assay

MMP was assessed using a JC-1 staining kit (Beyotime) according to the standard instructions of the kit. In short, HK-2 cells were pretreated as described above for 24 h, washed with PBS twice and plated in 6 wells. Subsequently, JC-1 dye was added to each well, and treated cells were then incubated in the dark at 37 °C for 20 min. After washing with cold JC-1 staining buffer, cultured cells were imaged by the ImageXpress® High Content Screening System (IXM; Molecular Devices LLC, Sunnyvale, CA, USA), and then the ratio of red/green fluorescence intensity was analysed.

### Co-immunoprecipitation experiment (Co-IP)

Treated cells were lysed with immunoprecipitation lysate buffer on ice for 30 min. After centrifugation at the highest speed for 30 min at 4 °C, the supernatants were obtained and then incubated overnight at 4 °C with HRP-anti-Myc (11814150001, Roche Applied Science, Madison, WI, USA), HRP-anti-Flag (M2, A8592, Sigma, St. Louis, MO, USA) and anti-HA-Ub antibodies with shaking followed by 4 h of incubation with protein A agarose beads. Next, the samples were centrifuged at 3000 rpm for 3 min at 4 °C, washed with immunoprecipitation buffer three times, and boiled for 5 min. Then, SDS–PAGE loading buffer was added to the beads, followed by analysis by western blotting.

### Statistical analysis

All experiments were performed with at least three biological replicates. All experimental data are expressed as the mean ± standard deviation (SD). A Student’s t test was utilized to analyse the difference between two groups, and one-way analysis of variance (ANOVA) was performed to evaluate the differences among at least 3 groups through GraphPad (Ver. Prism 7, GraphPad Prism Software, La Jolla, CA, USA). A P value less than 0.05 was considered statistically significant.

## Results

### ORN model mice exhibited increased renal lipid deposition and kidney injury

**Fig. 1:**
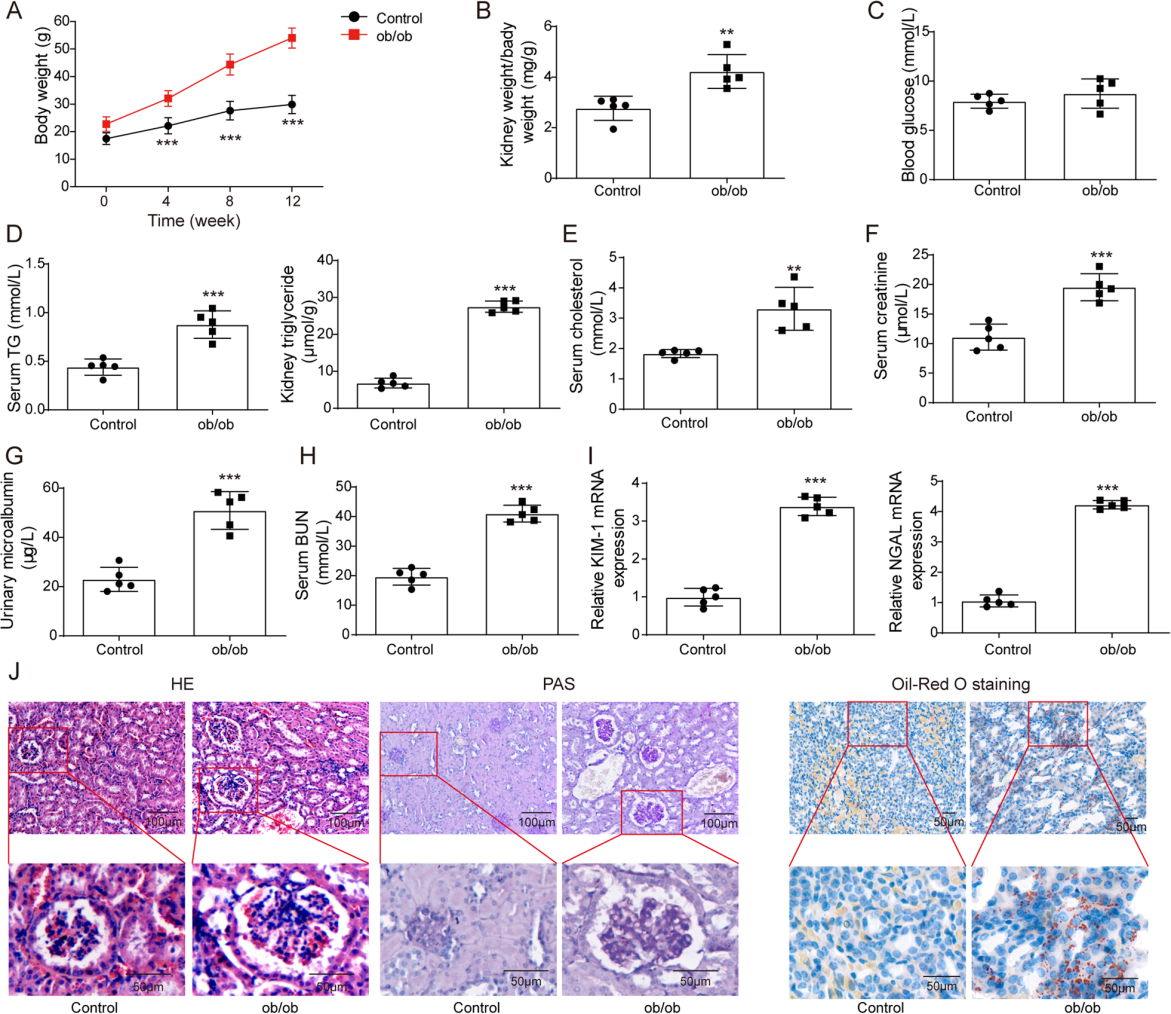
ORN model mice exhibited increased renal lipid deposition and kidney injury. **A**–**I** Body weights (**A**), kidney weight/body weight ratios (**B**), blood glucose levels (**C**), TG levels (**D**), serum cholesterol levels (**E**), serum creatine levels (**F**), urinary microalbumin levels (**G**), BUN levels (**H**), and KIM-1 and NGAL levels (**I**) of ORN model mice compared to control mice. **J** Kidney sections stained with HE, PAS and Oil Red O trichrome. ORN, obesity-related nephropathy; TG, triglyceride; BUN, blood urea nitrogen; KIM-1, kidney injury molecule-1; NGAL, neutrophil gelatinase-associated lipocalin; HE, haematoxylin and eosin; PAS, periodic acid-Schiff. Significance was assessed by a Student's t test. The experimental data are shown as the mean ± SD. N = 10. *P < 0.05, **P < 0.01, ***P < 0.001

To establish the ORN mouse model, we selected ob/ob mice and measured their body weights and kidney weight-to-body weight ratios. Compared with the C57BL/6 J mice in the control group, a significant increase in the body and kidney weights was observed in the ob/ob mouse group (Fig. 1A and B). Then, we tested the blood glucose levels, and the results showed that the blood glucose levels of ob/ob mice were higher than those of control mice, but not more than 16.7 mmol/L, indicating that ob/ob mice were determined to be simply obese mice rather than diabetic mice, which were used as an ORN mouse model for subsequent experiments (Fig. 1C). The TG levels in serum and kidney tissues of ORN model mice were increased compared with those of the control mice (Fig. 1D). Next, we measured more biochemical indicators in serum and urine samples. The results indicated that the serum cholesterol, serum creatine, urinary microalbumin, BUN, KIM-1 and NGAL levels of the ORN model mice were elevated significantly **(**Fig. 1E–I). Moreover, the kidney sections were stained with HE, PAS and Oil Red O, and then the histological pathology of the obese mice was observed under a microscope. The results from HE and PAS staining suggested that compared with the control mice, ORN model mice showed a significantly increased glomerular hypertrophy and mesangial matrix. As shown in the Oil Red O staining results, the accumulation of fat and large distended lipid droplets appeared in the kidneys of ORN model mice (Fig. 1J). These data showed that increased lipid deposition and kidney injury were observed in ORN model mice.

### Oxidative stress injury was observed in kidney tissues of ORN model mice

**Fig. 2:**
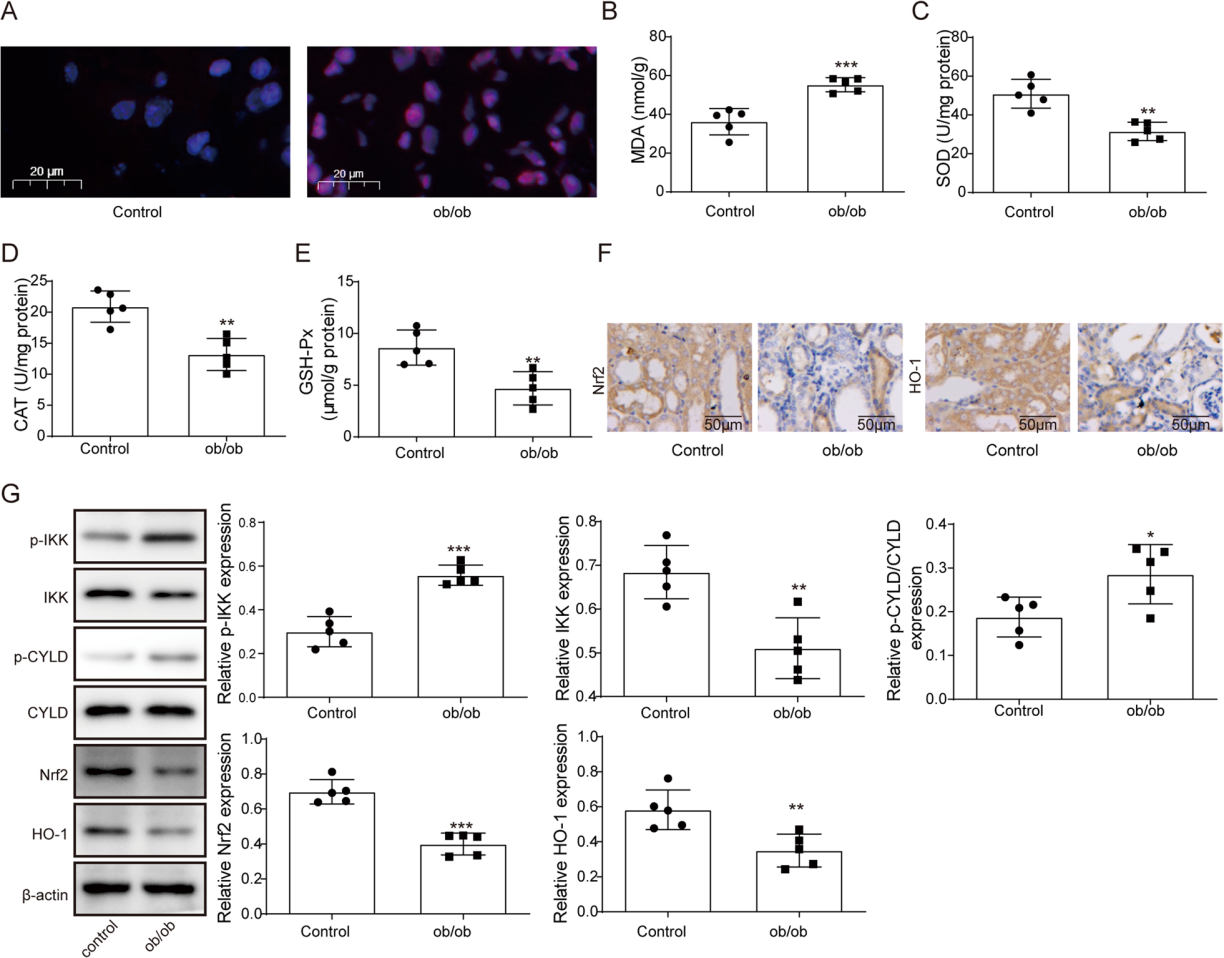
Oxidative stress injury was observed in kidney tissues of ORN model mice. **A** The production of ROS in the kidneys of ORN model mice and control mice was measured using DHE staining. **B**–**E** MAD level, SOD activity, CAT activity and GSH-Px activity were measured in mouse kidneys. **F** Representative IHC staining of Nrf2 and HO-1 in mouse kidneys. **G** Western blot analysis of p-IKK, IKK, p-CYLD, CYLD, Nrf2 and HO-1 expression in mouse kidneys. ORN, obesity-related nephropathy; ROS, reactive oxygen species; DHE, dihydroethidium; MAD, malondialdehyde; SOD, superoxide dismutase; CAT, catalase; GSH-Px, glutathione peroxidase; IHC, immunohistochemistry; Nrf2, NF-E2-related factor 2; HO-1, haem oxygenase 1; IKK, IκB kinase; CYLD, cylindromatosis. Significance was assessed by a Student's t test. The experimental data are shown as the mean ± SD. N = 10. *P < 0.05, **P < 0.01, ***P < 0.001

Based on the fact that oxidative stress is one of the main pathogenic mechanisms of ORN, we further investigated whether the kidney tissues of ORN model mice were damaged by oxidative stress. We first measured ROS production in the kidneys of the mice by staining with DHE, which is a ROS-sensitive vital dye. As shown in Fig. 2A, ROS production increased notably in the ORN model mouse group. Then, we assessed levels of MDA, a metabolic product of lipid peroxidation, as well as the activities of SOD, CAT, and GSH-px, which function as primary endogenous antioxidant enzymes. The results indicated that MDA levels increased while SOD, CAT, and GSH-px activities decreased in the kidneys of ORN model mice compared with the control mice **(**Fig. 2B–E). Nrf2 induces the expression of HO-1, thereby playing an antioxidant role in the kidney. We observed low expression levels of Nrf2 and HO-1 in the kidneys of ORN model mice by IHC and western blot (Fig. 2F and G). Taken together, these data suggested that oxidative stress injury occurred in the kidneys of ORN mice. Furthermore, we measured the protein expression of phosphorylated IKK (p-IKK), IKK, phosphorylated CYLD (p-CYLD) and CYLD in subsequent experiments. The results showed that p-IKK and p-CYLD expression was upregulated while Nrf2 and HO-1 expression was downregulated in the kidney tissues of ORN mice (Fig. 2G).

### IKK inhibitor alleviated lipid deposition and oxidative stress injury in HK-2 cells exposed to ox-LDL

**Fig. 3:**
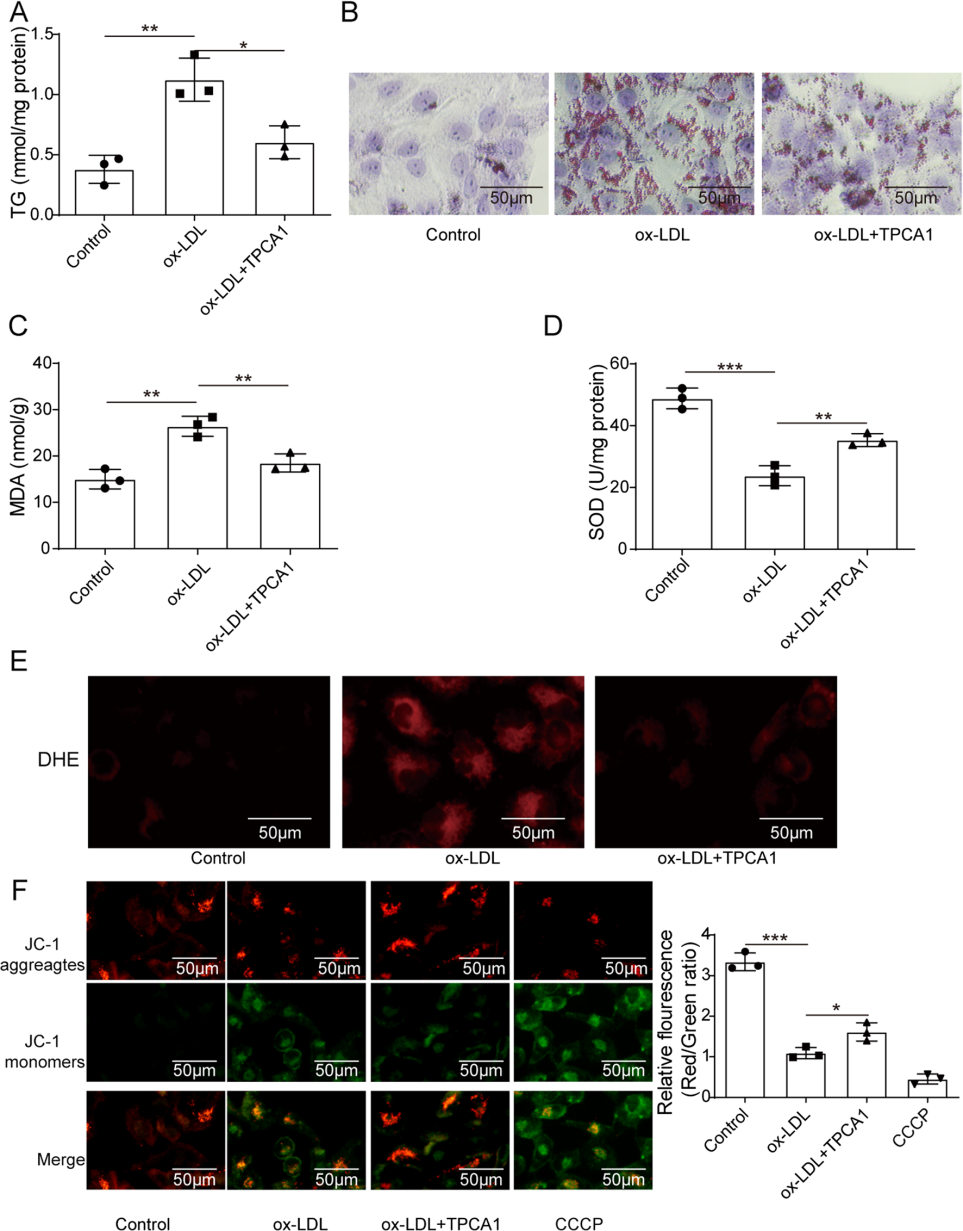
IKK inhibitor alleviated lipid deposition and oxidative stress injury in cells exposed to ox-LDL. HK-2 cells were exposed to ox-LDL to construct a cell model. TPCA1 acted as an IKK inhibitor. Cells were divided into three groups: control, ox-LDL, and ox-LDL + TPCA1. **A** TG levels of treated cells. **B** Representative Oil Red O staining of HK-2 cells in different groups. **C** and **D** qRT–PCR analysis of MAD levels and SOD activity in HK-2 cells in different groups. **E** DHE staining of intracellular ROS levels. **F** ΔΨm was measured using the JC-1 assay in treated HK-2 cells. IKK, IκB kinase; ox-LDL, oxidized low-density lipoprotein; HK-2, human kidney 2; TPCA-1, 2-[(aminocarbonyl)amino]-5-(4-fluorophenyl)-3-thiophenecarboxamide; TG, triglyceride; qRT–PCR, real-time quantitative reverse transcription PCR; MAD, malondialdehyde; SOD, superoxide dismutase; DHE, dihydroethidium; ROS, reactive oxygen species; ΔΨm, mitochondrial membrane potential. Significance was assessed by an ANOVA test. The experimental data are shown as the mean ± SD. N = 3. *P < 0.05, **P < 0.01, ***P < 0.001

HK-2 cells were used to perform in vitro experiments. HK-2 cells were incubated with ox-LDL to construct an ORN cell model. In vivo experiments showed that p-IKK was highly expressed in kidney tissues; therefore, ORN cells were exposed to TPCA1, which served as an IKK inhibitor, to further explore whether downregulation of IKK exerts effects on lipid deposition and oxidative stress injury in ORN cells. We first measured the TG levels of HK-2 cells in different groups, and the results showed that TG levels increased in the ox-LDL group compared with the control group, while the IKK inhibitor reduced TG levels to some extent (Fig. 3A). Oil Red O staining results then indicated that transfection with the IKK inhibitor also reduced the lipid deposition of ORN cells (Fig. 3B). Subsequently, we observed that the high level of MDA in ORN cells decreased while the low activity of SOD increased through silencing of IKK (Fig. 3C and D). Furthermore, ROS production in ORN cells was inhibited by transfection with TPCA1 (Fig. 3E). The main marker of mitochondrial injury is the change in mitochondrial membrane potential (ΔΨm) (Tang et al. 2016). Therefore, a JC-1 assay was used to measure HK-2 cell injury. Red fluorescence represents normal mitochondria, while green fluorescence represents ΔΨm depolarization. We found that ox-LDL-treated cells showed significantly enhanced green fluorescence, while IKK knockdown reversed this change, indicating that the IKK inhibitor alleviated ΔΨm (Fig. 3F). In conclusion, an IKK inhibitor protected HK-2 cells from ox-LDL-induced lipid deposition and oxidative stress injury.

### IKK promoted oxidative stress injury caused by obesity through CYLD phosphorylation

**Fig. 4:**
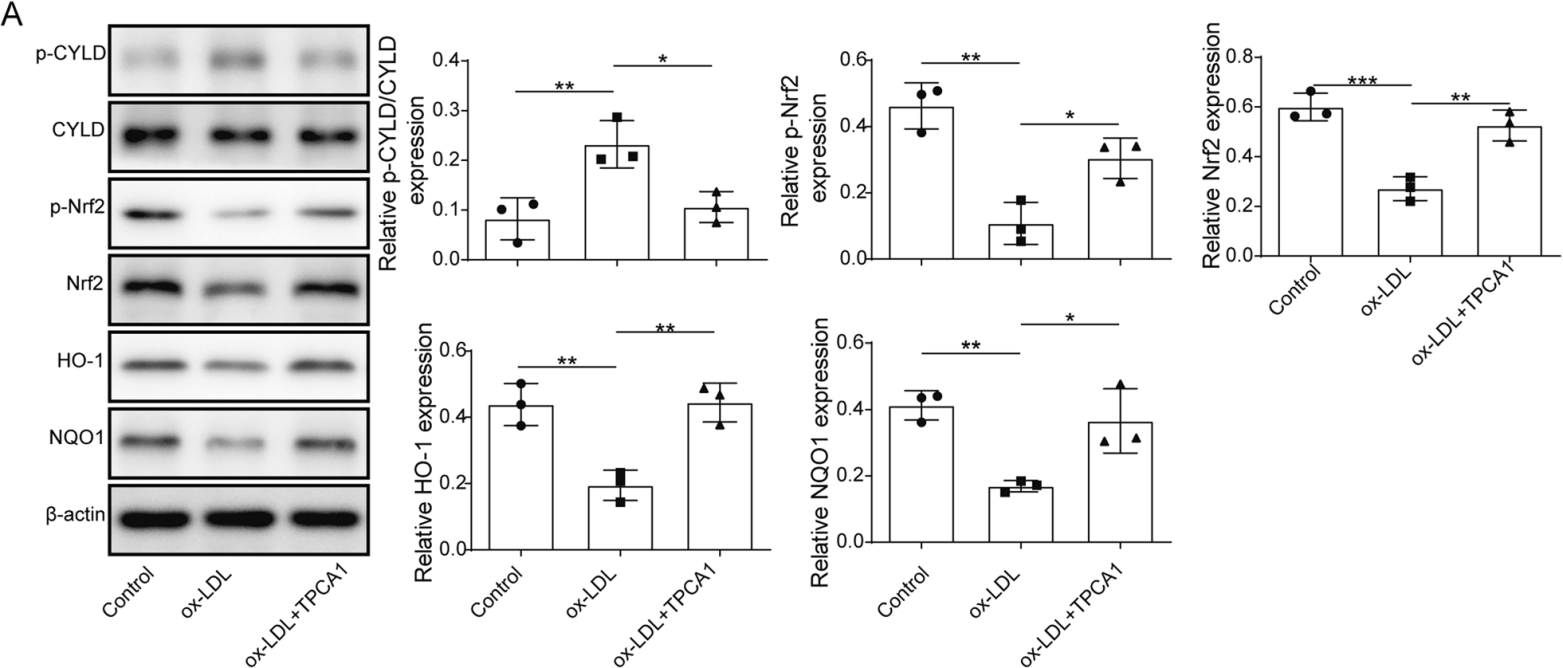
IKK promoted oxidative stress injury caused by obesity through CYLD phosphorylation. The protein levels of p-CYLD, CYLD, and p-Nrf2. Nrf2, HO-1 and NQO1 were measured using western blotting in treated HK-2 cells. IKK, IκB kinase; CYLD, cylindromatosis; Nrf2, NF-E2-related factor 2; HO-1, haem oxygenase 1; NQO1, NAD(P)H dehydrogenase quinone 1; HK-2, human kidney 2. Significance was assessed by an ANOVA test. The experimental data are shown as the mean ± SD. N = 3. *P < 0.05, **P < 0.01, ***P < 0.001

To further investigate how IKK causes oxidative stress injury, we examined the protein levels of CYLD in HK-2 cells based on the results of the in vivo experiments, which showed the increased expression of p-CYLD in ob/ob mice. The results showed that p-CYLD expression was significantly upregulated while p-Nrf2 expression was downregulated in ox-LDL-treated cells and that the expression of the antioxidant response elements (AREs) HO-1 and NQO1 was also downregulated, indicating that the phosphorylation of CYLD may promote oxidative stress injury in ORN cells. Notably, compared with the ox-LDL groups, p-CYLD showed low expression, while phosphorylated Nrf2 (p-Nrf2), HO-1 and NQO1 showed high expression in the ox-LDL + TPCA1 groups (Fig. 4). These findings suggested that IKK may promote oxidative stress injury by activating CYLD phosphorylation in the ORN.

### Knockdown of CYLD attenuated the effect of an IKK inhibitor on oxidative stress injury in ORN cells

**Fig. 5:**
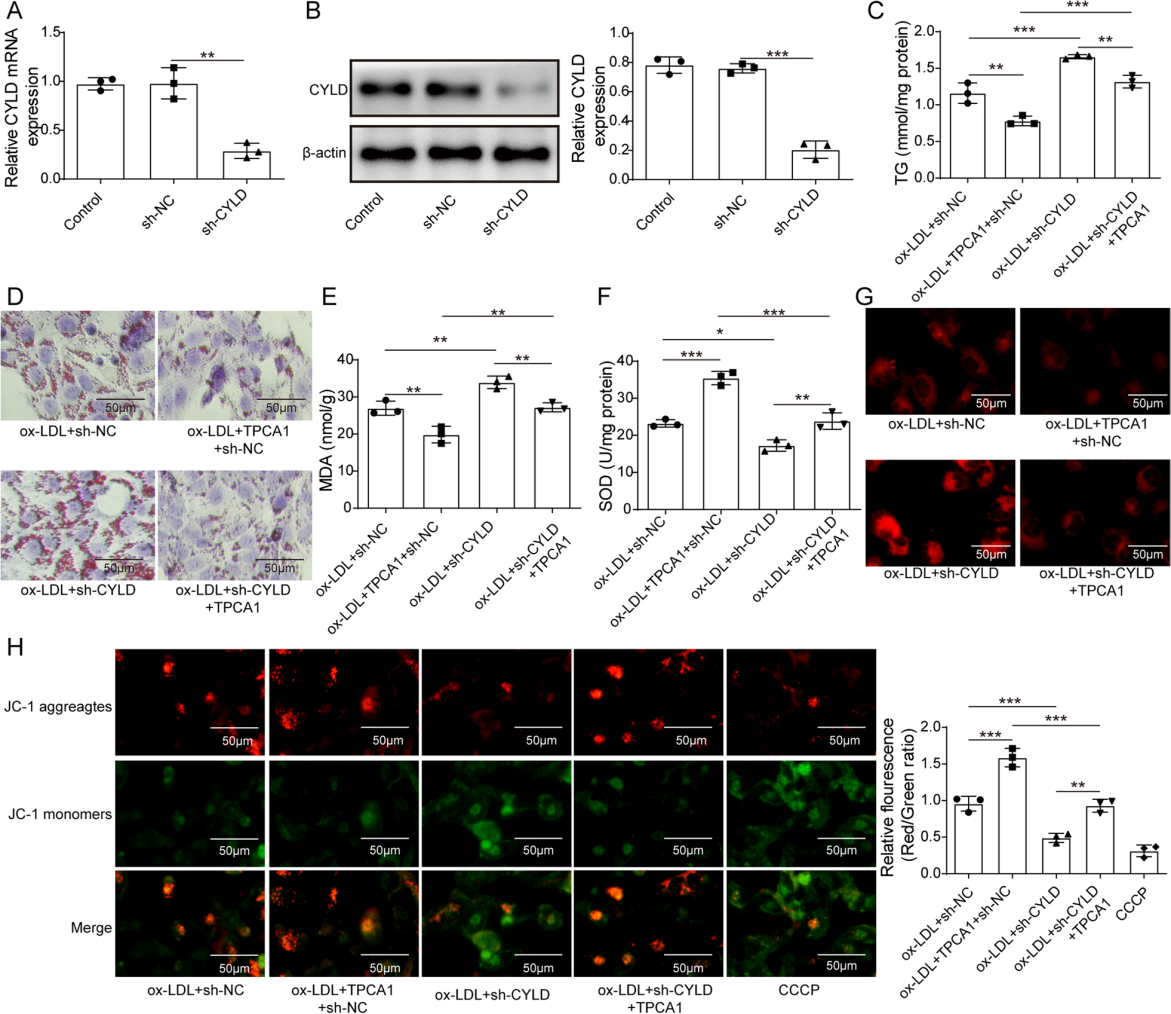
Knockdown of CYLD attenuated the effect of an IKK inhibitor on oxidative stress injury in an in vitro ORN model. Cells were transfected with sh-CYLD to silence CYLD expression and divided into four groups: ox-LDL + sh-NC, ox-LDL + TPCA1 + sh-NC, ox-LDL + sh-CYLD, and ox-LDL + TPCA1 + sh-CYLD. **A** and **B** The knockdown efficiency of sh-CYLD was assessed using qRT–PCR and western blotting. **C** Effect of CYLD knockdown on TG levels in treated HK-2 cells. **D** Lipid deposition in different groups was measured using Oil Red O staining. **E** and **F** MAD level and SOD activity of treated HK-2 cells. **G** and **H** ROS levels and ΔΨm of treated HK-2 cells were measured by DHE staining and JC-1 assay, respectively. CYLD, cylindromatosis; IKK, IκB kinase; ORN, obesity-related nephropathy; ox-LDL, oxidized low-density lipoprotein; NC, normal control; TG, triglyceride; HK-2, human kidney 2; MAD, malondialdehyde; SOD, superoxide dismutase; DHE, dihydroethidium; ROS, reactive oxygen species; ΔΨm, mitochondrial membrane potential. Significance was assessed by an ANOVA test. The experimental data are shown as the mean ± SD. N = 3. *P < 0.05, **P < 0.01, ***P < 0.001

To further verify the IKK/CYLD signalling axis in the ORN, we first measured the knockdown efficiency of sh-CYLD using qRT–PCR and western blotting. The results indicated that CYLD expression was downregulated in the sh-CYLD group (Fig. 5A and B). Then, the TG levels (Fig. 5C), lipid deposition (Fig. 5D), MDA levels (Fig. 5E), SOD activities (Fig. 5F), ROS production (Fig. 5G) and ΔΨm (Fig. 5H) of ORN cells were analysed after transfection with TPCA1, sh-CYLD or TPCA1 + sh-CYLD. The results suggested that compared with the control groups, knockdown of IKK inhibited TG levels, lipid deposition, MDA levels and ROS production while increasing SOD activities and ΔΨm. Notably, we observed opposite results in the ox-LDL + sh-CYLD groups. Interestingly, the results also suggest that compared with ox-LDL + TPCA1 + sh-NC groups, the TG levels, lipid deposition, MDA levels and ROS production in ox-LDL + TPCA1 + sh-CYLD groups did not decrease significantly, indicating that the inhibitory effect of IKK inhibitor on oxidative stress injury of ORN cells was suppressed by knockdown of CYLD.

### The deubiquitinase CYLD reduced the ubiquitination of Nrf2

**Fig. 6:**
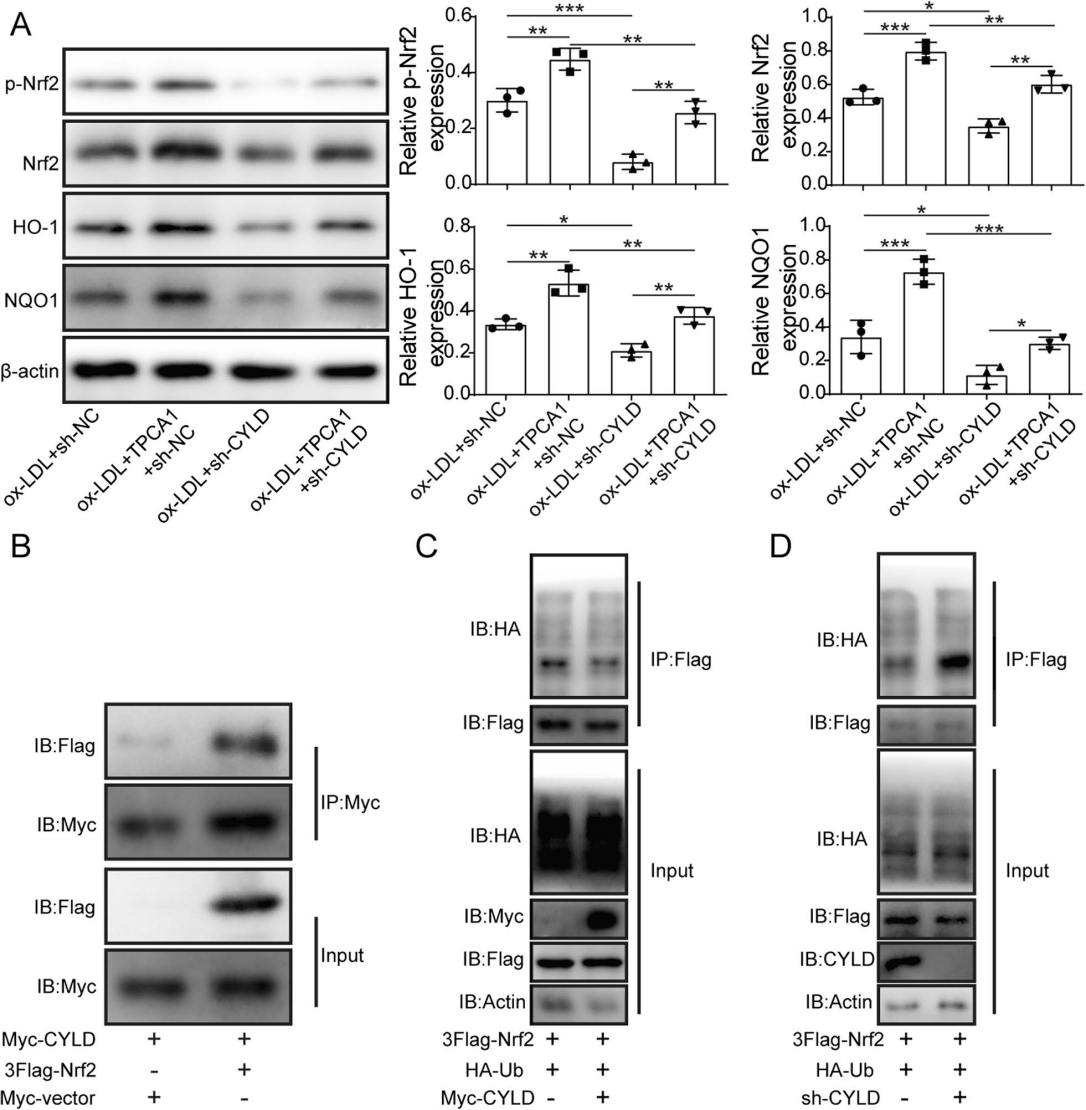
The deubiquitinase CYLD reduced the ubiquitination of Nrf2. **A** The protein expression levels of p-Nrf2, Nrf2, HO-1 and NQO1 in treated HK-2 cells. **B** The interaction between CYLD and Nrf2 was analysed using Co-IP. **C** and **D** Co-IP was performed to assess the effect of CYLD on the ubiquitination of Nrf2. CYLD, cylindromatosis; Nrf2, NF-E2-related factor 2; HO-1, haem oxygenase 1; NQO1, NAD(P)H dehydrogenase quinone 1; HK-2, human kidney 2; Co-IP, co-immunoprecipitation. Significance was assessed by an ANOVA test. The experimental data are shown as the mean ± SD. N = 3. *P < 0.05, **P < 0.01, ***P < 0.001

Next, we began to investigate the downstream molecules of the IKK/CYLD signalling axis. Previous results had shown that Nrf2 expression was downregulated in both ORN mice and cells and that an IKK inhibitor increased Nrf2 expression in vitro (Figs. 2G and  4). Nrf2 functions as an important regulator of cellular defence mechanisms against oxidative stress (Bellezza et al. 2018). We therefore speculated that Nrf2 may be affected by the IKK/CYLD pathway, thereby promoting oxidative stress injury in ORN cells. As shown in Fig. 6A, the IKK inhibitor upregulated the expression of p-Nrf2, Nrf2, HO-1 and NQO1, while knockdown of CYLD reversed this effect. Subsequently, co-immunoprecipitation (co-IP) assays were performed on extracts of HK-2 cells transfected with 3 × Flag-tagged Nrf2 and Myc-tagged CYLD to analyse the molecular interaction between Nrf2 and CYLD. The results suggested that Nrf2 directly bound to CYLD (Fig. 6B). Moreover, Nrf2 exhibited a series of high-molecular-weight species in ORN cells transfected with sh-CYLD, indicating that Nrf2 was ubiquitinated by knockdown of CYLD (Fig. 6C–D**)**. These findings suggested that the deubiquitination effect of CYLD was inactivated, leading to the ubiquitination of Nrf2, which further promoted oxidative stress injury in ORN cells.

### MG132 inhibited the promoting effect of CYLD knockdown on oxidative stress in ORN cells

**Fig. 7:**
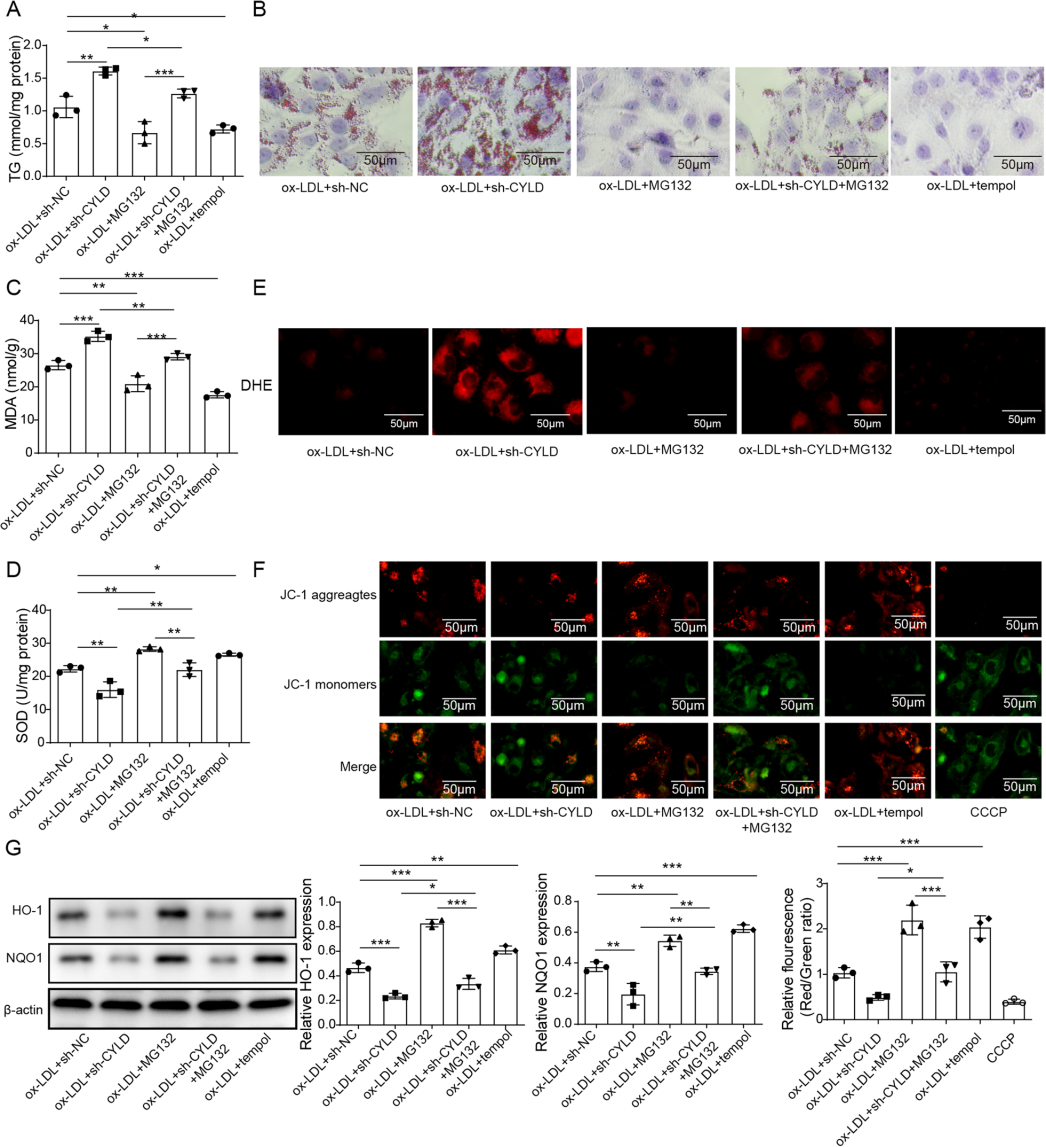
MG132 inhibited the promoting effect of CYLD knockdown on oxidative stress in ORN cells. The proteasome inhibitor MG132 was used to regulate the expression of Nrf2. Cells were divided into five groups: ox-LDL + sh-NC, ox-LDL + sh-CYLD, ox-LDL + MG132, ox-LDL + sh-CYLD + MG132 and ox-LDL + tempol. **A**–**F** The effect of MG132 on TG levels, lipid deposition, MDA levels, SOD activity, ROS levels and ΔΨm. **G** The protein expression of HO-1 and NQO1 was measured using western blotting. CYLD, cylindromatosis; ORN, obesity-related nephropathy; Nrf2, NF-E2-related factor 2; ox-LDL, oxidized low-density lipoprotein; NC, normal control; MAD, malondialdehyde; SOD, superoxide dismutase; DHE, dihydroethidium; ROS, reactive oxygen species; ΔΨm, mitochondrial membrane potential; HO-1, haem oxygenase 1; NQO1, NAD(P)H dehydrogenase quinone 1. Significance was assessed by an ANOVA test. The experimental data are shown as the mean ± SD. N = 3. *P < 0.05, **P < 0.01, ***P < 0.001

Previous studies have reported that the proteasome inhibitor MG132 protects tissues and cells against oxidative injury by activating the Nrf2/ARE pathway (Duan et al. 2011; Dreger et al. 2009; Sahni et al. 2008). MG132 cells were therefore incubated with ORN cells to further verify the interaction between CYLD and Nrf2. Cells were cotreated with ox-LDL + sh-NC, ox-LDL + sh-CYLD, ox-LDL + MG132, ox-LDL + sh-CYLD + MG132 or ox-LDL + tempol. As shown in Fig. 5, knockdown of CYLD reduced SOD activities and ΔΨm in ORN cells, increased TG levels, lipid deposition and MDA levels, and facilitated ROS production. However, we observed that these effects were reversed by cotreatment with MG132 or tempol in vitro (Fig. 7A–F). In addition, HO-1 and NQO1 expression was increased after treatment with MG132 or tempol in CYLD-silenced ORN cells (Fig. 7G). In conclusion, the activation of the Nrf2/ARE signalling pathway suppressed the promoting effect of CYLD silencing on oxidative stress.

## Discussion

Although oxidative stress has been extensively studied as a major pathogenic mechanism in a variety of diseases, including ORN (D'Agati et al. 2016), further investigation is needed on how oxidative stress occurs and how to mitigate oxidative stress injury in ORN. Weight loss, RAAS (renin–angiotensin–aldosterone system) blockade and improvement of insulin resistance can relieve ORN to some extent, which also has many limitations (Kambham et al. 2001; Xu et al. 2017; Saiki et al. 2005; Yang et al. 2020). Therefore, it is still necessary to explore more effective therapeutic targets for ORN. In this study, our data suggested that IKK induced phosphorylation of CYLD and then inactivated its deubiquitination activity. Furthermore, we observed that CYLD reduced the ubiquitination of Nrf2; thus, phosphorylated CYLD instead promoted the ubiquitination of Nrf2, eventually leading to oxidative stress injury in the ORN. These findings suggested that the IKK/CYLD/Nrf2 axis might serve as a novel target for mitigating oxidative stress injury in ORN.

It is well known that obesity causes organ damage by inducing oxidative stress, and previous studies have suggested that ROS production is one of the driving factors for the development of nonalcoholic fatty liver disease into nonalcoholic steatohepatitis (Pierantonelli and Svegliati-Baroni 2019). This second-hit hypothesis has also been reported in kidney injury. For example, Andres-Hernando et al. suggested that increased oxidative stress is related to worse renal tubular injury (Andres-Hernando et al. 2019). This was consistent with our findings that oxidative stress injury occurred in both in vivo and in vitro ORN models. In addition, we also found that IKK inhibitors could reduce lipid deposition and oxidative stress injury, which might be a potential specific mechanism of oxidative stress causing kidney damage in ORN. IKK is a kinase complex that phosphorylates the NF-κB inhibitory protein IκBα, which facilitates ubiquitination of IκBα and subsequent proteasomal degradation (Hayden and Ghosh 2004). It is therefore easy to speculate whether IKK mediates the phosphorylation of other proteins or enzymes. As a recognized tumour suppressor gene, CYLD has been extensively studied in many cancers, and it has been found that CYLD is phosphorylated in various cancer cells through IKK induction (Hutti et al. 2009; Xu et al. 2020). This is consistent with our experimental results. In this study, the IKK inhibitor TPCA1 was used to measure the possible involvement of IKK in mediating CYLD phosphorylation in the ORN. We observed increased phosphorylation of CYLD in ORN model cells, while IKK blockade reactivated CYLD and reduced phosphorylation. Moreover, we found that TPCA1 decreased TG levels, MDA levels and ROS production and increased SOD activities and ΔΨm, indicating that oxidative stress injury in ORN cells was relieved. Therefore, we hypothesized that IKK promoted oxidative stress injury caused by obesity by stimulating CYLD phosphorylation.

**Fig. 8:**
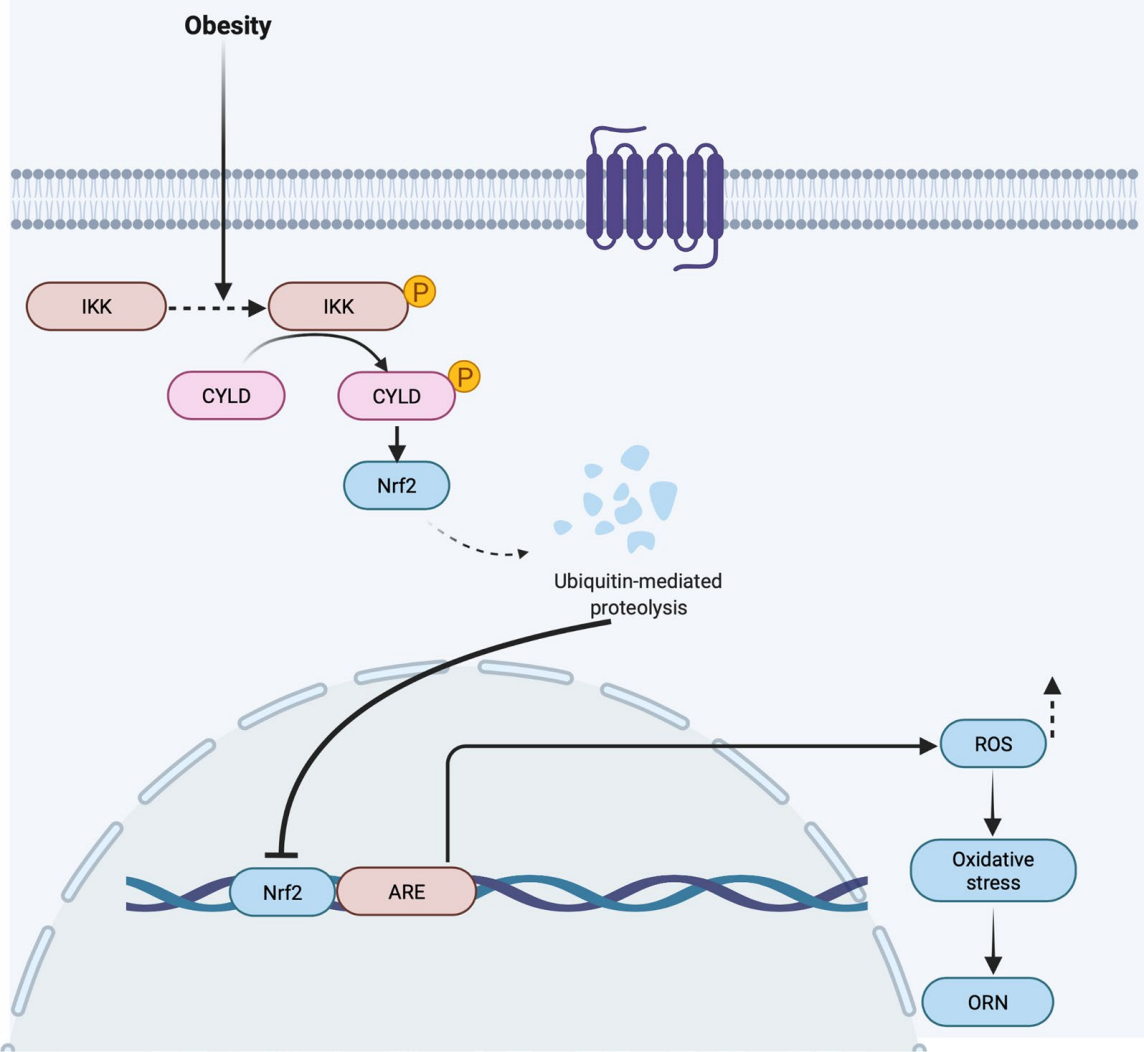
Schematic representation of the proposed mechanism by which the phosphorylation of IKK and CYLD mediated the activation of the Nrf2/ARE pathway, thereby inducing oxidative stress in human kidney cells. The imbalance between the increase in ROS and/or the decrease in antioxidant activity promotes oxidative stress injury to tissues or cells. Regulation of mitochondrial production of ROS is required for the proper adaptation of renal cells to metabolic stress. The phosphorylation of IKK induced phosphorylation of CYLD results in the inactivation of its deubiquitination activity and is thus unable to prevent the ubiquitination of Nrf2, which promoted the production of ROS and the depolarization of mitochondrial membrane potential and ultimately facilitated oxidative stress injury in vitro, which is conducive to the development of ORN

To further confirm this speculation, we began to explore the downstream molecules of CYLD. Our findings indicated not only the phosphorylation of CYLD but also the low expression of Nrf2 and HO-1 both in vitro and in vivo, which suggested that Nrf2 might be involved in the development of ORN. Increasing studies have shown that Nrf2 exerts protective effects by ameliorating the production of ROS and increasing the antioxidant capacity (Abreu et al. 2015; Ma 2013; Hybertson et al. 2011). We therefore speculated whether the phosphorylation of CYLD affected the antioxidant capacity of Nrf2. The results suggested that Nrf2 directly bound to CYLD. Moreover, Co-IP results indicated that Nrf2 was ubiquitinated by knockdown of CYLD. Thus, it is conceivable that CYLD could exert a deubiquitinating effect on Nrf2 under normal conditions. A logical explanation of these findings is that the phosphorylation of CYLD results in the inactivation of its deubiquitination activity and is thus unable to prevent the ubiquitination of Nrf2. Taken together, we concluded that IKK promoted the ubiquitination of Nrf2 by activating the phosphorylation of CYLD, which further aggravated the oxidative stress injury of ORN (Fig. 8).

However, the conclusion needs to be supported by more in vivo data, which will be the focus of our future work. Additionally, hypertension occurs in more than 65% of obesity cases (Hall et al. 2015), which has been proven to be a necessary factor for obesity-induced renal injury in vivo (do Carmo et al. 2009). Moreover, azilsartan medoxomil was reported to show a protective effect on the kidney by lowering blood pressure in obese rats (Khan et al. 2014). Taken together, these conclusions have shown the important role of hypertension in obesity-induced renal injury; however, whether there is a synergistic action between hypertension and lipid deposition has not been reported, which may be another research direction for our study.

## Conclusion

In conclusion, our findings demonstrated that Nrf2 was inhibited in ORN, resulting in oxidative stress injury. An IKK inhibitor could reduce the phosphorylation of CYLD and inhibit the ubiquitination of Nrf2 to improve oxidative stress-induced kidney injury in ORN. This observation provides a feasible basis for relieving oxidative stress injury in ORN.

## Data Availability

The datasets used or analyzed during the current study are available from the corresponding author on reasonable request.

## Abbreviations

ORN: Obesity-related nephropathy
ROS: Reactive oxygen species
ARE: Antioxidant response element
Nrf2: NF-E2-related factor 2
HO-1: Haem oxygenase 1
DUBs: Deubiquitinating enzymes
CYLD: Cylindromatosis
IKK: IκB kinase
DMEM: Dulbecco’s modified Eagle's medium
FBS: Foetal bovine serum
qRT–PCR: RNA extraction and quantitative real-time PCR
SDS–PAGE: Sodium dodecyl sulfate polyacrylamide gel electrophoresis
PVDF: Polyvinylidene difluoride
PAS: Periodic acid-Schiff
DHE: Dihydroethidium
IHC: Immunohistochemistry
MMP: Mitochondrial membrane potential
Co-IP: Co-immunoprecipitation
SD: Standard deviation
TG: Triglyceride
BUN: Blood urea nitrogen
KIM-1: Kidney injury molecule-1
NGAL: Neutrophil gelatinase-associated lipocalin
HE: Haematoxylin and eosin
MAD: Malondialdehyde
SOD: Superoxide dismutase
CAT: Catalase
GSH-Px: Glutathione peroxidase
ox-LDL: Oxidized low-density lipoprotein
HK-2: Human kidney proximal tubular epithelial cells
qRT–PCR: Real-time quantitative reverse transcription PCR
NQO1: NAD(P)H dehydrogenase quinone 1
TPCA-1: 2-[(Aminocarbonyl)amino]-5-(4-fluorophenyl)-3-thiophenecarboxamide
